# Secretive and close? How sharing secrets may impact perceptions of distance

**DOI:** 10.1371/journal.pone.0282643

**Published:** 2023-04-26

**Authors:** Mariela E. Jaffé, Maria Douneva, Elianne A. Albath

**Affiliations:** 1 Center for Social Psychology, University of Basel, Basel, Switzerland; 2 University Psychiatric Clinics, Basel, Switzerland; 3 Freelancer, Berlin, Germany; University of Hertfordshire, UNITED KINGDOM

## Abstract

Having secrets is incredibly common. However, secrecy has only recently started to receive more attention in research. What has largely been neglected are the consequences of secret-sharing for the relationship between sharer and receiver; a gap we aim to fill in this project. Previous research has shown that closeness can make secret-sharing more likely. Building on research from the self-disclosure and relationship literature, we experimentally investigate in three studies (*N* = 705) whether confiding a secret to somebody might in turn increase perceptions of closeness. In addition, we test whether the valence of the secrets moderates the hypothesized effect. While confiding negative secrets might signal a high level of trust and lead to a similar closeness as confiding positive secrets, they might also present a burden to the receiver and lead to a different pattern of closeness. To provide a holistic picture, we build on a variety of methods and investigate three perspectives: Study 1 focused on the receiver and showed that another person sharing secrets (vs. nonconfidential information) decreased the distance in the eyes of the receiver. Study 2 tested how an observer perceives the relationship between two people. Distance was judged to decrease when secrets (vs. nonconfidential information) were shared, however, this difference was not significant. Study 3 tested whether lay theories about sharing secrets predict behavior, and how sharing information may be used to change perceived distance on the receiver’s side. Participants preferred to share neutral compared to secret information and positive compared to negative secrets irrespective of the distance condition. Our results contribute to the understanding of how sharing secrets affects the way individuals think about each other, how close they feel to each other, and how they interact with each other.

## Introduction

We all have our personal secrets; stories from the past or present that we do not want to share with other people. In his famous novel *Ulysses*, James Joyce reflects on this fact and points out that “secrets, silent, stony sit in the dark palaces of both our hearts” [1]. Secrets have not only sparked the interest of writers, but also of scientists. Researchers have defined secrecy as “an intention to conceal information from one or more individuals” [2] and note that it is incredibly common. Slepian and colleagues [2] found that almost all of their study participants reported currently having at least one secret. These secrets often concerned extra-relational thoughts, sexual behavior, lies, and romantic desires not shared with anybody, but also abortion, sexual orientations, and marriage proposals, which are not kept entirely to oneself but are not openly shared either.

Turning back to Ulysses, James Joyce does not only observe that we all have secrets, but describes individuals as “weary of their tyranny” and secrets as “tyrants willing to be dethroned” [1]. His observation is in line with research showing that there is a tension between revealing and concealing private information [3]. Having secrets can result in negative consequences for the secret-keeper: Keeping a secret is associated with rumination due to suppression of thoughts and more intrusive thinking [4, 5], it can be stressful, and can harm health; for an summary, see [6]. Furthermore, it is not necessarily only the concealment of secrets, but also the associated increase in experiencing fatigue [7] and more frequent spontaneous thoughts about the secrets that negatively impact individuals’ well-being [2, 8].

Researchers have also investigated the antecedents and consequences of “dethroning the weary tyrants” [1] by sharing a secret with another person (e.g., by confiding in a friend or family member). Deciding to share a secret is not as uncommon as one might think: Sharing a secret with at least one or two persons seems to be at least as common as not sharing a secret at all [2]. It appears that although there are reasons to keep a secret, there are also reasons for sharing it.

Previous research has provided some explanations for why individuals might want to share information and secrets. One line of research investigates the general tendency to share personal information and self-disclose to another person or organize thoughts by using expressive writing [9, 10]. Another line of research more specifically focuses on secret sharing [6, 11]. Both lines of research have much in common, however, sharing a secret may go beyond mere self-disclosure, as it can be described as “a more profound social act, as it entails honest and selective disclosure of something specifically intended to be kept from others” [11]. Moreover, different from disclosing personal information, disclosing secrets is typically associated with a request for help [12].

Revealing personal information, even if only in writing [9, 10], seems advantageous. Disclosing personal information may ease worry [13]. Turning more specifically to secrets, revealing secrets may decrease distress when experiencing intrusive thoughts [5], increase self-esteem [14] and well-being [15]. Being asked to conceal information is associated with elevated levels of discomfort and tension [16]. Another line of research has investigated a different perspective, namely the impact of sharing personal information or a secret on the receiver. Receivers may experience increased intimacy, but at the same time a burden and negative feelings while guarding those secrets [17]. The consequences may depend on the size and severity of the secrets: The secret’s importance, its negative valence, and the negative face threat of secret-keeping (e.g., as being asked to keep a secret leads to behavioral constraints) are all positively associated with cognitive burden for the receiver [18].

Another line of research aims to integrate both perspectives, namely those of the secret-keeper and the secret-receiver, and points out that there are circumstances under which the secret-keeper is better off keeping the secret to him- or herself; for an overview, see [19]. Critical to this could be the recipient’s reaction towards the disclosure of the secret: Afifi and Caughlin [14] showed that rumination among individuals who reveal a secret is only reduced when they perceive the receiver’s reaction as positive.

As outlined above, previous research has primarily focused on the impact of disclosing information or secrets on the secret-sharer or on the receiver individually. However, the disclosure of a secret is a social encounter and involves at least two people, it cannot exist in isolation. It therefore appears plausible that not only the individuals, but also the relationship between sharer and receiver should be affected by the exchange of the secret. Still, this aspect has often been neglected. In this article, we aim to fill this gap by focusing on the effects of sharing secret information about oneself on the perceived social distance between secret-sharers and receivers. To this end, we investigate potential changes in the relationship from three perspectives, implementing a variety of methods: First, we aimed to understand the *receiver’s* perspective when being told secrets of positive and negative valence compared to nonconfidential information using audio recordings of phone calls as well as asking for participants’ experiences (Study 1). Next, we aimed to understand how an *observer* would judge the effects of secret-sharing on the relationship between sharer and receiver using written vignettes (Study 2). Both studies allowed us to investigate individuals’ lay theories, meaning individuals’ assumptions about “the nature of the self and the social world” [20]. Individuals might use lay theories to derive hypotheses to predict, but also to understand and interpret their social world in an efficient way [21]. Going beyond the interpretation of the social world, lay theories impact individuals’ perceptions and actions [21], more specifically in social relationships [22]. In the final study, we therefore aimed to understand how *sharers* might deliberatively choose to share (negative or positive) secrets in cases where they want to create social closeness or distance between themselves and another person (Study 3). This approach also allowed us to investigate whether the lay theories examined in the study on the observer’s perspective might transfer to individuals’ actual choices and behavior when sharing information. Predictions and expectations of actors and observers might be asymmetric [23], meaning that people who self-disclose fears, anxieties, or vulnerabilities might overestimate the recipient’s negative reaction [24, 25]. Observers might therefore have a different take on the impact of sharing secrets on the social distance between interaction partners than the secret-sharers themselves.

In addition to looking at the effects of secret-sharing from these three perspectives, we also used a variety of methods to cross-validate our findings, as “each type of method, considered alone, is imperfect” [26] and implementing different methods increases confidence in and the generalizability of our findings. To this end, we used audio recordings to assess individuals’ reactions in a setting with higher ecological validity, which we complemented by asking for descriptions of personal experiences (Study 1). Furthermore, we included written vignettes, which increased the internal validity of our study due to the higher level of standardization. Last, we investigated goal-directed behavior to understand not only individuals’ perceptions but also their actions when sharing secrets. All in all, this work provides an integrative view on how secret-sharing affects the relationship between individuals.

### Social distance and sharing secrets

Sharing secrets can mean different things: A person may decide to share a secret by *confessing* to the person whom the secret was originally kept from [6]. Alternatively, a person may share their secret with somebody else who may not be involved in the secret, thereby *confiding* in them [6, 11]. Here, we focus on the latter case, and by sharing a secret, we refer to the act of confiding in someone and not necessarily confessing something to them.

An action such as confiding a secret presumably interplays with social distance between the sharer and the receiver. Social distance is a measure of intimacy between two people, meaning that it reflects how close or distant they feel to each other [27, 28]. While a person’s best friend is socially very close, a stranger is socially distant. Previous research has already identified social distance as an antecedent of revealing secrets, which will be discussed below. However, the consequences of revealing secrets on social distance between interaction partners are less clear and require further research. These consequences will therefore be the focus of our research article.

So far, a few studies have established the link that psychological closeness may lead to the disclosure of personal information in general, and eventually also to the disclosure of secrets. Jourard and Friedman [29] found that participants disclosed more information as an experimenter reduced the distance to participants by self-disclosing personal information (which could also be interpreted as reducing social distance) as well as establishing minimal physical contact. These early findings are corroborated by more recent ones, showing that the closer the individuals’ social network ties, the more secrets were confided in them [12]. Apparently, individuals are more likely to reveal secrets to people to whom they have close social ties, meaning to people who are spatially and socially close to them. Furthermore, being compassionate and assertive predicts having secrets confided in oneself [12] and one could speculate that compassion could signal psychological proximity.

### Would confiding secrets impact social distance?

As outlined above, confiding in psychologically close others seems more likely than revealing secrets to psychologically distant others or strangers. The link between closeness and confiding secrets, however, does not need to be unidirectional, meaning that revealing secrets may also impact perceptions of psychological closeness, see [30]. Previous research investigating self-disclosure indicates that sharing personal information—which can be but is not necessarily secret—is important for developing closeness and gaining emotional support in relationships [31, 32]. This is also the core assumption of Social Penetration Theory [33], which provides a framework describing the development of interpersonal relationships, with the metaphor of an “onion model” [34]. More specifically, the theory proposes that layers of personal information are “peeled back” over time [34] by increasing disclosure [32] and that this self-disclosure increases intimacy to a certain point [34]. Furthermore, it has been found that sharing extraordinary compared to ordinary experiences enhances closeness, but that discomfort associated with the interaction is necessary for this effect to occur [35]. The act of sharing a secret could involve both: sharing something extraordinary, and experienced discomfort while sharing.

Interestingly, self-disclosure can also serve as a form of boundary regulation, as individuals control how much (or how little) contact they maintain with others [36]. As described before, self-disclosure can be used to develop closeness in a relationship; see also research on the experimental generation of interpersonal closeness [37, 38]. By communicating in a warm way with distant others, distance can be bridged and perceived closeness increased [39]. On the other hand, if individuals decide to not share or to even actively hide information, they might create (physical) distance between themselves and other persons by avoiding contact with them [36].

Going beyond self-disclosure, the disclosure of one’s secrets may signal trust and the need for social support [11]. Confiding a secret in someone may, similarly to self-disclosure and disclosure of the interaction partner [40], foster the development of a closer relationship, which may result in perceptions of reduced social distance. Based on this research, we hypothesize that social distance between a sharer and receiver is not only a predictor, but also a consequence of sharing secrets.

### Importance of the research question

Many, if not all of us, carry one or more secrets. Some of them may prove to be rather insignificant if others would find out about them, whereas others might profoundly change the way others perceive us (e.g., when confessing infidelity towards one’s partner). Whether or not one should share a secret with someone is a question that research has often examined from the individual’s point of view (e.g., will the secret-sharer ruminate less after telling a friend?). However, as individuals usually share secrets with close others who they are likely to interact with in the future, it is critical to take into account how people expect their relationship with the other person to be influenced by the act of sharing a secret. Individuals’ beliefs and expectations regarding this change in the relationship most probably influence whether they themselves are willing to trust and confide in somebody else.

As our three studies focus on different perspectives, our results not only contribute to the understanding of individuals’ lay theories with regards to secret-sharing or secret-receiving, but eventually also allow us to make predictions about the way individuals think about each other, how close they feel toward each other, and how they will eventually interact in the future after one has shared a secret with the other. At the same time, our research directly addressed gaps in the information-sharing literature that have recently been voiced, for example, under which conditions sharing positive information with others leads to positive consequences [41], or whether people can accurately predict consequences of sharing information with others [41].

### Proposed hypotheses and research plan (preregistered)

Our overall hypothesis is that the revelation of secrets impacts perceptions of change in social distance between the secret-sharer and the receiver. More specifically, we predict that sharing secrets (compared to nonconfidential information) about oneself *decreases* perceived social distance between the two interaction partners (Hypothesis 1).

We also investigate, in an exploratory manner, whether the valence of the information shared might make a difference for the receiver (Hypothesis 2). Previous research has shown that it is not the intention but the outcome of a behavior that impacts distance perceptions [42], which leads to the speculation that the hypothesized decrease in social distance depends on the nature of the revealed secrets (the “outcome”). Vrij, Paterson, Nunkoosing, Soukara, and Oosterwegel [43] cautioned against making strong claims about the benefits of disclosure without acknowledging that disclosing information can also be associated with negative aspects. Zhang and Dailey [18] showed that the valence of the shared secret is associated with relational distancing initiated by the receiver of the secret. Sharing positive personal information may result in feelings of closeness and therefore bridge psychological distance. However, sharing a “dark” secret about oneself might result in negative reactions on the side of the receiver and therefore, in turn, result in negative consequences for the sharer [14]. In consequence, the receiver and sharer could decide to increase social (or more general: psychological) distance between themselves to reduce the intensity of negative affect [44]. In contrast, sharing a “dark” secret may signal higher levels of trust and vulnerability to the receiver, and this type of self-disclosure could then result in higher levels of liking [45] and thereby enhance social closeness. We therefore test for differential effects of positive and negative secrets. As derived from previous literatures and outlined above, sharing a negative secret could a) have the same effect as sharing a positive secret, namely to decrease social distance, b) have an even stronger effect than sharing a positive secret, namely to decrease social distance even more, or c) be associated with an *in*crease in perceived social distance between interaction partners. Each one of those outcomes would be of interest to better understand effects of valence when sharing secrets.

We tested our assumptions by taking three perspectives in three studies. Throughout the studies, we compared a secret condition to a nonconfidential condition. To ensure comparability between the conditions’ content and to rule out alternative explanations for our findings (such as that effects are driven by previous levels of closeness or different information shared), we used content-wise identical information in both conditions but either framed it as secret or as general news. In Study 1, participants were at the receiver’s end of the sharing process, and were asked to listen to an audio recording presented as a phone call in which one person (i.e., the secret-sharer) tells them a secret or news about him-/herself. The audio recording contained a brief description of the situation and the content of the sharer’s revelation, and participants were subsequently asked to evaluate their relationship with the secret-sharer. Furthermore, Study 1 exploratorily tapped into participants’ own experiences by asking them to recall a situation in which somebody shared a secret (vs. news) with them and whether this sharing was associated with a change in distance between them. In Study 2, participants took the perspective of an external observer by reading a brief scenario in which two other people exchanged information. After reading the scenario, they were asked to evaluate the perceived distance between these two people. As Study 2 focused on individuals’ lay theories, we then tested whether lay theories might also impact participants’ choices and behaviors when sharing a secret themselves. In Study 3, participants were therefore invited to the lab and put into the position of a secret-sharer, where we aimed to understand what type of information they shared when their objective was to decrease versus increase social distance.

### Initial support from an informal pretest

We conducted a pretest to gain preliminary insight and support for our main hypothesis that confiding secrets decreases perceived social distance. In this pretest, we asked participants to take the receiver’s position and read a vignette in which a woman shares a piece of information about a new job offer with them (translated Vignette 1 in S1 File, reframed as a story that Helen tells the participant). As we did in all studies, we presented all participants with the same information, but we either clearly framed it as a secret in the secret condition or news in the nonconfidential condition. Participants were then asked to imagine the situation and reflect on the information by describing where the situation might have taken place and how they would react towards the information. Subsequently, all participants rated whether they would feel relaxed versus tense, well versus unwell, secure versus insecure, happy versus sad, and satisfied versus dissatisfied (semantic differential; 5-point scale) in the situation. Participants were then asked to evaluate the imagined social distance between themselves and the woman in the vignette on the Inclusion of Other in the Self Scale [46]. This scale served as a pictorial measure of closeness ranging from 1 = *interpersonally distant* to 7 = *interpersonally close*. We recruited 103 German-speaking participants via Clickworker for this pretest. Prior to data analysis, we excluded two participants who asked for their data to be excluded or reported insufficient language proficiency. We excluded seven further participants when screening their descriptions of the scene as two answers were not readable or not in German and five other answers were identical and not informative for the scenario, giving us reason to believe that one person completed the survey several times. The dataset, analysis script, and original materials can be found online: https://osf.io/m68sr/.

The resulting sample consisted of 38 female and 56 male participants (*M*_*age*_ = 41.67; *SD*_*age*_ = 13.09). The descriptive results indicate that participants in the secret condition (*n* = 48) compared to participants in the news condition (*n* = 46) imagined feeling more relaxed (*M* = 2.69 vs. *M* = 2.91), well (*M* = 2.23 vs. *M* = 2.46), more secure (*M* = 2.29 vs. *M* = 2.50), happier (*M* = 2.31 vs. *M* = 2.43), and more satisfied (*M* = 2.04 vs. *M* = 2.26). Most importantly, participants in the secret condition imagined feeling closer to the person in the vignette compared to participants in the news condition (*M* = 4.85 vs. *M* = 4.30). For a better overview, all results are summarized in Table 1.

**Table 1:** Overview of results from pretest.

	Condition			
Dependent Variable	Secret	News	*p*-value (two-sided)	*d*
	*M* (*SD*)	*M* (*SD*)		
Reaction towards information shared				
… relaxed (1) versus tense (5)	2.69 (1.26)	2.91 (1.19)	.374	0.18
… well (1) versus unwell (5)	2.23 (1.10)	2.46 (1.17)	.333	0.20
… secure (1) versus insecure (5)	2.29 (1.17)	2.50 (1.28)	.411	0.17
… happy (1) versus sad (5)	2.31 (1.13)	2.43 (1.31)	.629	0.10
… satisfied (1) versus dissatisfied (5)	2.04 (0.82)	2.26 (0.86)	.209	0.26
Evaluation of distance				
… interpersonally distant (1) to close (7)	4.85 (1.53)	4.30 (1.58)	.089	-0.35

These descriptive differences do not reach statistical significance (presumably due to the lack of power to detect a small effect of *d* = -.35). We believe that for the main studies it is therefore important to consider the effect size by not only increasing the sample size but also by increasing the number of trials and asking participants to rate more than one situation.

The main purpose of the pretest was to provide us with critical insights regarding the potential direction of the effect, which is in line with our assumptions, and regarding the design of the subsequent studies. It is noteworthy that we observed a pattern in line with our assumptions despite the hypothetical nature of the study (i.e., participants neither recalled real instances nor did they interact with a real person, instead they only imagined a situation). All in all, the descriptive differences provided a promising outlook with regard to the three preregistered and conducted studies by lending initial support to our main hypothesis.

## Study 1

Study 1 investigated whether a person sharing information about her- or himself that is either framed as a secret or not impacts participants’ perceptions of social distance between them and the person. To this end, participants in Study 1 were asked to listen to audio recordings that were presented as phone calls. All audio recordings included a person describing a situation in which they told the participants a piece of information that was, at the end, framed as a secret or not. This information was furthermore either positive or negative. Participants were asked to carefully listen to these audio recordings and to subsequently judge potential changes in social distance between themselves and the person sharing the information with them. After evaluating all vignettes, we asked them to recall a situation in which somebody shared a secret (news) with them and how this incident impacted social distance.

Study 1 allowed us to test Hypotheses 1 and 2: Exchanging a secret reduces perceptions of social distance more than exchanging a piece of news (Hypothesis 1), but possibly to a different extent in the case of positive compared to negative secrets (Hypothesis 2). The setup allowed us to test our hypotheses in both an experimental setup with standardized material (audio recordings which are content-wise identical between conditions) and more ecologically valid material (idiosyncratic experiences but also materials that mimic interpersonal everyday exchanges).

### Methods

Approval by the University’s IRB (#20-18-1) was obtained for all of the studies. Participants provided written consent within the studies. We report all measures, manipulations, and exclusions.

#### Participants

The study was conducted as an online study advertised as a *study on the evaluation of behavior* via the online participant recruitment platform Prolific. Our a priori power analysis with an α-level of .05, a desired power of .95, and a small to medium effect size estimate (*f* = 0.15) while assuming a small correlation between repeated measures (.20) indicated a required sample size of 280 participants [47, 48]. We increased this number by 10% (while ensuring that cell numbers were balanced) to reach the required sample size even if participants needed to be excluded due to the criteria described below. The aspired sample therefore comprised 308 participants.

As prescreening criteria, we required all participants to speak English as their native language and to be using headphones throughout the study. Participants who did not give consent were screened out from the survey. Additionally, eligible participants were asked to indicate whether they saw any reason as to why their data should not be used for statistical analyses at the end of the study. If they asked for exclusion, we did not use their data for the analysis. We analyzed complete data sets only.

Three hundred eighty-eight participants started the study, of which 310 provided written informed consent and completed the study and received £1.40 as compensation. When looking at participants who indicated reasons for exclusion, we excluded four participants because they indicated having difficulties with the scales or the audio recordings, having misread questions, or not having answered questions correctly. Even though we set English as the native language as a prescreening criterion on Prolific, 17 participants indicated that they spoke English fluently, but that it was not their native tongue. We excluded these participants from the data analysis as well, which resulted in a final sample of 289 participants (156 female, 130 male, three undisclosed, *M*_*age*_ = 33.21, *SD*_*age*_ = 12.09). Participants were randomly assigned to four between-subjects conditions with 73 participants listening to positive secrets, 70 to negative secrets, 74 to positive information, and 72 to negative information.

#### Design

Study 1 was set up as a mixed design with two between-subjects factors (secrecy: message framed as secret vs. not, and valence: information is positive vs. negative) and one within-subjects factor (audio recording number: one to five). Judgments of change in social distance between the participant and the person speaking in the audio recording, as well as between the participants and the person who had really told them secrets or news in the past served as dependent variables and were measured with two different approaches (see Materials below).

#### Materials

All five stories in the audio recordings concerned situations in which one person shared a piece of information about him- or herself with the participants (adjusted versions of the vignettes presented in S1 and S2 Files, see pretest), with this information being either framed as secret or not. This information was furthermore either positive (see S1 File) or negative (see S2 File). All vignettes were designed to cover the main categories of secrets identified in previous research [2] and were balanced in regards to names of the protagonists and the degree of control concerning the focal topic. Furthermore, the recordings were obtained from five different speakers, who each recorded one negative and one positive vignette to keep voices constant across conditions.

All audio recordings were presented in a randomized order. To assess our key dependent variables, it was important that we could differentiate between social distance per se and the variable of interest, namely perceptions of *change* in social distance between participants and the person in the audio recordings. To this end, we first asked all participants “After having listened to the message, do you perceive that your relationship might have changed to some extent?” (answer options ranging from 1 = *not at all;* 7 = *very much*). If participants indicated a change (values greater than 1), we then asked “After having listened to the message, do you perceive your relationship as closer or more distant than before?” (1 = *closer than before*; 7 = *more distant than before; direct assessment*). Furthermore, we used the IOS scale [46], which served as a pictorial measure of interpersonal closeness between two individuals, and asked participants to evaluate the imagined social distance that would best describe the relationship. The scale consisted of 7 images, ranging from the largest distance between two circles at the left side (coded as 1) to the highest overlap and closeness on the right side of the screen (coded as 7).

To further minimize the risk of demand characteristics affecting our dependent variables, we also asked participants to rate aspects of the exchange that were not related to social distance, such as the length of the conversation (1 = *very short*; 7 = *very long*), how soon the individuals would meet again (1 = *very soon*; 7 = *in a long while*), and whether the next encounter would take place in a more private or more public place (1 = *very public place*; 7 = *very private place*).

To assess our key dependent variables in regards to participants’ own past experiences, we asked them to recall a situation in which a person shared either negative or positive secrets versus news with them (in line with one of the four conditions they had been assigned to). We asked them to describe the situation without necessarily revealing the content of the information. Next, we asked participants: “After that person told you this, did you perceive that your relationship might have changed to some extent?” (1 = *not at all;* 7 = *very much*). If participants indicated a change (values greater than 1), we then asked “Did you perceive your relationship to be closer or more distant than before the person told you this?” (1 = *closer than before*; 7 = *more distant than before*).

#### Procedure

Participants were welcomed to the study about “evaluation of behavior”. At the very beginning, we explained that they needed to use headphones during the study. To ensure that they met the study’s requirements, they listened to a recording of a person describing a still life picture, and were then asked to select all objects from a list of five that had been mentioned in the description. If participants correctly identified the objects mentioned, they could carry on with the study. After providing informed consent, they then learned that the study aimed to understand evaluations of other people’s behavior. To this end, they were asked to imagine being on the phone with a friend and to then listen to the audio recordings. In this call, the friend described a situation and shared some information with them. After listening to each recording, participants were asked to rate how confidential the content of the exchange was (which served as a manipulation check) on a seven-point Likert scale (1 = *not confidential at all*; 7 = *very confidential*). Participants first rated whether they perceived a change in the relationship, and if so, they then rated the change in perceived social distance between themselves and the person, as well as the additional measures described above before listening to the second recording and so forth.

After having read and evaluated all recordings, participants were asked to think about a past experience in which somebody shared a negative / positive piece of information / secret with them. Again, participants rated whether they perceived a change in the relationship, and if so, rated the change in perceived social distance.

Last, participants provided demographic information (gender and age) and were asked whether they saw any reasons as to why their data should not be included in the study. We also asked them if they had an assumption about what the underlying research question in the study might have been. At the end of the study, individuals were debriefed and thanked for their participation.

### Results

All analyses were conducted with RStudio (Version number: 2022.07.01) [49]. This also applies to the subsequent studies.

#### Preregistered data analysis: Confidentiality of information

After excluding all participants according to our a priori defined criteria (see above), we used a repeated measures ANOVA to analyze whether our sample perceived recordings in the secret condition as more confidential than the recordings in the nonconfidential condition. This analysis served as a check to test whether our manipulation of secrecy was successful. Looking at means averaged across scenarios, the content shared was generally rated as rather confidential, but more so in the secret condition compared to the not secret condition; *M* = 5.92, *SD* = 0.86 versus *M* = 5.27, *SD* = 1.11, *F*(1, 287) = 30.59, *p* < .001, η_p_^2^ = .10. This main effect was not qualified by an interaction between secrecy and scenario number. As several assumptions of the ANOVA were violated, we used the WRS2 package [50] to compute a robust test for our hypothesis (not preregistered). In line with the ANOVA, there was a significant main effect of secrecy, *Q*(1, 149.99) = 20.66, *p* < .001, but unlike in the ANOVA, it was qualified by an interaction with scenario (*Q*(4, 118.93) = 3.96, *p* = .005, indicating that the effects of secrecy differed in strength for the different scenarios, see Table 2). The main effect of scenario was not significant, *Q*(4, 118.93) = 1.39, *p* = .240.

**Table 2:** Overview of means (and standard deviations) from Study 1 without listwise deletion (see ANOVA analysis above).

Dependent variable	Condition		Scenario					
	Secrecy	Valence	1	2	3	4	5	Average
Confidentiality	Secret	Collapsed	5.78 (1.45)	5.71 (1.38)	5.98 (1.22)	6.08 (1.22)	6.03 (1.33)	5.92 (0.86)
	Non-secret	Collapsed	5.40 (1.80)	5.27 (1.71)	5.25 (1.60)	5.13 (1.90)	5.30 (1.62)	5.27 (1.11)
Extent of change	Secret	Positive	3.21 (1.83)	3.33 (1.83)	3.47 (1.92)	2.97 (1.89)	3.55 (2.14)	3.30 (1.59)
		Negative	4.56 (1.93)	5.47 (1.53)	2.99 (1.77)	3.80 (2.20)	5.24 (1.49)	4.41 (1.26)
	Non-secret	Positive	3.23 (1.72)	3.45 (1.80)	3.46 (1.87)	2.91 (1.72)	4.04 (1.95)	3.42 (1.39)
		Negative	4.40 (1.74)	4.99 (1.60)	2.81 (1.86)	3.54 (1.90)	4.65 (1.76)	4.08 (0.99)
Change in social distance	Secret	Positive	3.52 (1.36)	2.73 (1.20)	2.95 (1.30)	3.04 (1.04)	2.60 (1.26)	2.95 (0.88)
		Negative	3.05 (1.70)	4.70 (1.74)	2.96 (1.25)	2.00 (1.22)	4.91 (1.39)	3.80 (1.12)
	Non-secret	Positive	4.22 (1.18)	3.34 (1.53)	2.93 (1.23)	3.28 (1.16)	2.73 (1.39)	3.29 (1.00)
		Negative	3.12 (1.76)	4.77 (1.56)	3.53 (1.46)	2.62 (1.47)	5.12 (1.36)	3.98 (1.12)
IOS scale	Secret	Positive	4.00 (1.45)	4.70 (1.36)	4.71 (1.46)	4.38 (1.34)	5.18 (1.24)	4.63 (1.16)
		Negative	4.39 (1.69)	3.33 (1.72)	4.40 (1.47)	5.34 (1.19)	2.81 (1.62)	3.87 (1.32)
	Non-secret	Positive	3.27 (1.42)	4.83 (1.48)	4.84 (1.31)	4.06 (1.29)	4.63 (1.63)	4.34 (1.24)
		Negative	4.33 (1.76)	2.97 (1.63)	4.17 (1.45)	5.18 (1.52)	2.39 (1.49)	3.63 (1.28)
Personal experience: change in social distance	Secret	Positive	-	-	-	-	-	2.51 (1.14)
		Negative	-	-	-	-	-	4.24 (2.11)
	Non-secret	Positive	-	-	-	-	-	2.57 (1.16)
		Negative	-	-	-	-	-	3.56 (1.91)
Personal experience: IOS scale	Secret	Positive	-	-	-	-	-	5.32 (1.53)
		Negative	-	-	-	-	-	3.88 (2.11)
	Non-secret	Positive	-	-	-	-	-	5.39 (1.25)
		Negative	-	-	-	-	-	3.97 (2.20)
Additional variables	Secrecy	Valence	1	2	3	4	5	Average
Length of conversation	Secret	Positive	2.68 (1.03)	3.11 (1.17)	3.36 (1.14)	2.96 (1.07)	3.20 (1.25)	3.00 (0.84)
		Negative	2.52 (0.94)	2.63 (1.17)	2.79 (1.05)	3.21 (1.23)	2.21 (0.92)	2.56 (0.87)
	Non-secret	Positive	2.76 (1.30)	3.15 (1.27)	3.67 (1.10)	3.09 (1.31)	3.42 (1.46)	3.11 (1.14)
		Negative	2.88 (1.29)	2.94 (1.10)	3.11 (1.20)	3.24 (1.22)	2.77 (1.24)	2.91 (1.03)
Expected next meeting	Secret	Positive	3.79 (1.51)	2.93 (1.17)	3.33 (1.12)	3.60 (1.23)	2.80 (1.21)	3.26 (0.86)
		Negative	3.10 (1.71)	4.46 (1.64)	3.04 (1.11)	2.28 (1.23)	4.63 (1.51)	3.71 (1.10)
	Non-secret	Positive	4.27 (1.36)	2.61 (1.36)	3.10 (1.29)	3.17 (1.42)	2.88 (1.35)	3.20 (1.00)
		Negative	2.57 (1.53)	4.59 (1.57)	3.04 (1.16)	2.04 (1.04)	4.83 (1.21)	3.52 (0.85)
Location of next encounter	Secret	Positive	3.71 (1.37)	4.09 (1.42)	4.53 (1.44)	4.09 (1.36)	4.92 (1.18)	4.25 (0.91)
		Negative	4.71 (1.74)	3.87 (1.80)	4.40 (1.38)	5.70 (1.32)	3.57 (1.63)	4.24 (1.12)
	Non-secret	Positive	3.46 (1.38)	4.10 (1.51)	4.64 (1.32)	3.89 (1.33)	4.80 (1.31)	4.17 (0.90)
		Negative	5.25 (1.67)	3.73 (1.72)	4.09 (1.38)	5.89 (1.12)	3.05 (1.60)	4.32 (1.02)

#### Preregistered data analysis: Change in social distance (direct assessment)

**Fig 1:**
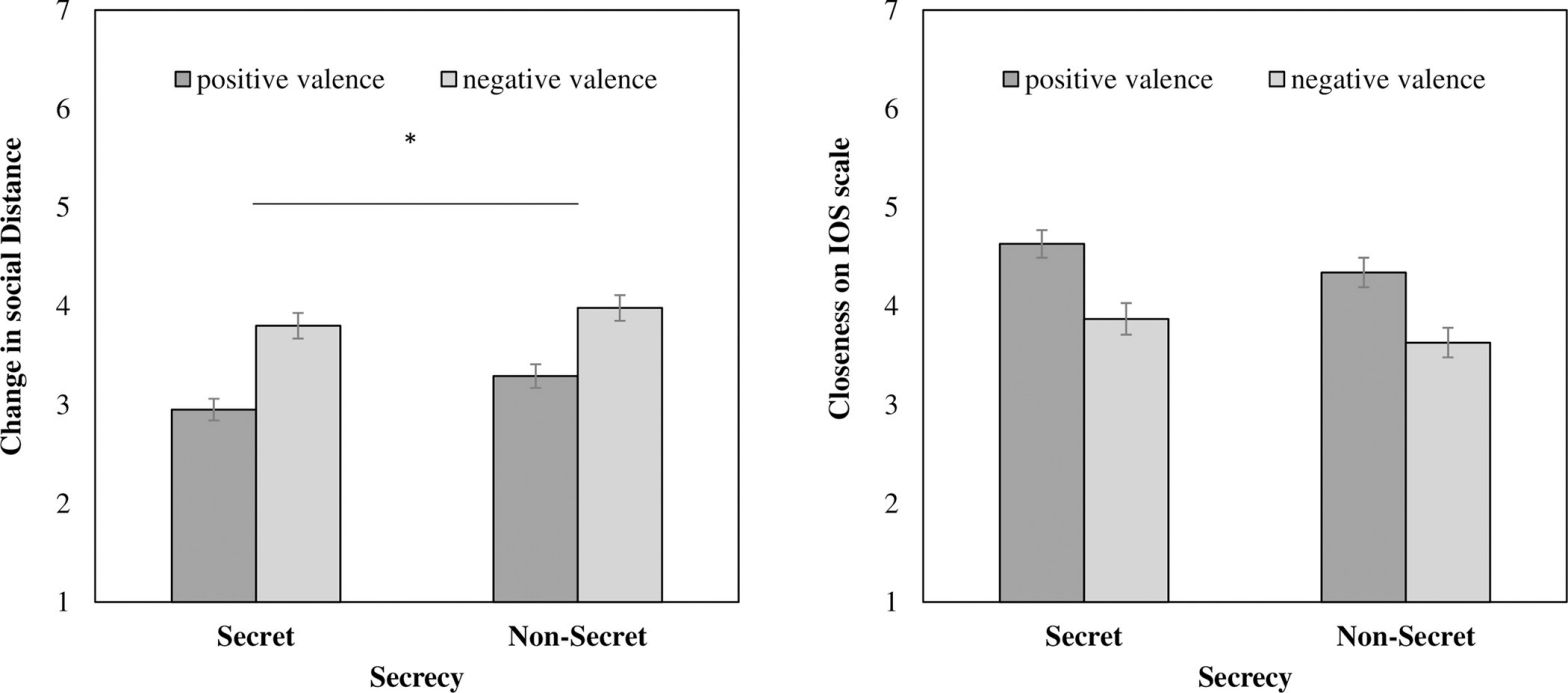
Effects of secrecy and valence on change in social distance (left) and on closeness on the IOS scale (right) in Study 1. Change in social distance and IOS closeness ratings range from 1 (closer than before / largest distance between circles) to 7 (more distant than before / largest overlap between circles). Error bars represent standard errors.

To test Hypotheses 1 (main effect of secrecy) and 2 (interaction between secrecy and valence), we calculated a repeated measures ANOVA with number of audio recordings (scenario) as a repeated measure and secrecy as well as valence as between-subjects factors. Perceptions of change in social distance served as a continuous dependent variable. We calculated this analysis once for the direct evaluation of social distance in the relationship and once for the IOS scale measure (for all recordings in which participants indicate that a change has occurred). As the repeated measures ANOVA applies listwise deletion of all participants with one missing value, participants who had indicated no change for any one of the five scenarios and therefore had one or more missing values for social distance evaluations were excluded from the analysis. This was true for 121 participants, which means that running the repeated measures ANOVA reduced the sample to 168 participants. Results for the direct evaluation of change in social distance indicated the hypothesized significant main effect of secrecy, *F*(1, 164) = 8.71, *p* = .004, η_p_^2^ = .05, showing that participants indicated feeling closer to the person than before (means < 4), but more so in the condition where the information was framed as secret compared to not secret, *M* = 3.22, *SD* = 0.76 versus *M* = 3.56, *SD* = 0.91, see Fig 1.

Results further yielded an unexpected significant main effect of valence, *F*(1, 164) = 9.80, *p* = .002, η_p_^2^ = .06, indicating that distance decreased more in the positive compared to the negative valence condition, *M* = 3.20, *SD* = 0.82 versus *M* = 3.57, *SD* = 0.84. We further found a significant effect of scenario, *F*(3.76, 615.97) = 20.80, *p* < .001, η_p_^2^ = .11, indicating that distance decreased more or less depending on the scenario (collapsed across valence conditions, i.e., different information is grouped together, which is why we do not further analyze specific scenario effects). The main effect of valence was qualified by a significant interaction between valence and scenario, *F*(3.76, 615.97) = 52.39, *p* < .001, η_p_^2^ = .24. Descriptive results showed that distance decreased more in the positive compared to the negative condition for scenarios 2, 3, and 5, whereas the opposite pattern occurred for scenarios 1 and 4. In contrast to our second hypothesis, there was no interaction between secrecy and valence, *F*(1, 164) = 0.00, *p* = .963, η_p_^2^ = .00, nor any other interaction effects, all *F*s < 1.63. As several assumptions of the ANOVA were violated, we used the WRS2 package [50] to compute a robust test for the between-condition effects (not preregistered). Results were in line with the ANOVA, showing significant main effects of secrecy (*Q* = 4.95, *p* = .029) and valence (*Q* = 9.11, *p* = .004), but no secrecy-valence interaction (*Q* = .019, *p* = .892).

#### Preregistered data analysis: Change in social distance (pictorial assessment)

Assessing perceptions of distance with the IOS-scale yielded similar patterns. The repeated measures ANOVA indicated a non-significant effect of secrecy, *F*(1, 164) = 1.10, *p* = .295, η_p_^2^ = .01. The descriptive result patterns indicated more closeness (more overlap between the circles) in the secret compared to the not secret condition, *M* = 4.33, *SD* = 1.13 versus *M* = 4.18, *SD* = 1.10. We further found a main effect of valence, *F*(1, 164) = 6.91, *p* = .009, η_p_^2^ = .04, showing significantly more closeness in the positive compared to negative valence condition, *M* = 4.47, *SD* = 1.10, and *M* = 4.04, *SD* = 1.11. The analysis also replicated the effect of scenarios, *F*(3.79, 622.19) = 18.24, *p* < .001, η_p_^2^ = .10, which was qualified by an interaction between scenarios and valence, *F*(3.79, 622.19) = 67.36, *p* < .001, η_p_^2^ = .29. Descriptive results for scenarios 2, 3, and 5 showed that closeness was rated more highly in the positive compared to negative condition, whereas the opposite pattern occurred in scenarios 1 and 4. The interaction between secrecy and valence was again non-significant, *F*(1, 164) = 0.01, *p* = .930, η_p_^2^ = .00, which is also true for the remaining interaction effects, all *F*s < 1.73. As assumptions of the ANOVA were violated, we used the WRS2 package [50] as a robust test for the between conditions effects (not preregistered). Results were in line with the ANOVA, showing a non-significant main effect of secrecy (*Q* = 0.21, *p* = .648), a significant main effect of valence (*Q* = 8.76, *p* = .004), and no significant secrecy-valence interaction (*Q* = 0.00, *p* = .951).

#### Preregistered data analysis: Personal experiences

To test our hypotheses with participants’ personal experiences, we calculated an ANOVA with secrecy and valence as between-subjects factors. Perceptions of change in social distance served as a continuous dependent variable. Two hundred twenty-two participants (i.e., 77% of the sample) indicated that a change had occurred after the information or secret had been shared. Results from the ANOVA then showed a significant main effect of valence, *F*(1, 218) = 35.50, *p* < .001, η_p_^2^ = .14, indicating that participants felt closer in the positive than in the negative condition, *M* = 2.54, *SD* = 1.14 versus *M* = 3.89, *SD* = 2.03. We did not find a main effect of secrecy (Hypothesis 1), *F*(1, 218) = 2.30, *p* = .131, η_p_^2^ = .01, or a significant interaction (Hypothesis 2), *F*(1, 218) = 2.75, *p* = .099, η_p_^2^ = .01. We again computed a robust analysis [50] (not preregistered) that corroborated previous results by indicating a significant main effect of valence (*Q* = 30.91, *p* = .001), a non-significant effect of secrecy (*Q* = 2.83, *p* = .097), and a non-significant secrecy-valence interaction (*Q* = 3.27, *p* = .075). When excluding participants that had not described a suitable situation in the open textbox (i.e., 9 participants = 3%; not preregistered), the results remained stable. Similarly, using the ratings on the IOS-scale instead of the Likert scale did not change the pattern of results. For this pictorial measure, we found a significant main effect of valence, *F*(1, 218) = 33.44, *p* < .001, η_p_^2^ = .13, which again showed that participants felt closer (more overlap) in the positive than in the negative condition, *M* = 5.36, *SD* = 1.38 versus *M* = 3.92, *SD* = 2.15. Neither the main effect of secrecy nor the valence-secrecy interaction was significant, all *F*s < 0.11. Results were again corroborated when using a robust analysis [50], indicating a significant main effect of valence (*Q* = 29.04, *p* = .001), no significant main effect of secrecy (*Q* = 0.00, *p* = .962), and no interaction effect (*Q* = 0.06, *p* = .813). Results further proved stable when excluding participants without a suitable situation (not preregistered, see above).

#### Preregistered data analysis: Identification of research question

We also analyzed how many participants had correctly guessed our research question and whether excluding them affected our results. We asked an independent research assistant to read through participants’ open answers. When asked, 104 participants (i.e., 36%) thought that the study was related to secrets and/or interpersonal relations (however, this might simply reflect that they paid attention to the instructions throughout the study). Excluding such a large part of our sample in addition to participants who had missing values on the main dependent variables (due to listwise deletion) resulted in a remaining sample of 104 participants and a reduction of our effects. When rerunning the repeated measures ANOVAs on perceptions of distance (both directly assessed and measured via the IOS scale), we found no main effect of secrecy. The main effects of valence and scenario remained significant and were qualified by the previously found interaction between valence and scenario. Using robust analyses [50] corroborated the ANOVA results.

#### Not preregistered additional data analysis

As 121 participants were excluded from our main analysis due to the listwise deletion procedure when computing repeated measures ANOVAs, we reran the analysis using linear mixed models [51] using the lme4 and lmerTest package [52, 53]. Using the rstatix-package [54], we excluded extreme outliers (three data points for ratings regarding closeness and none for the IOS scale) before computing the analysis and used effect coding for secrecy and valence (-0.5 for *secrets*, 0.5 for *information*; -0.5 for *positive valence*, 0.5 for *negative valence*). We included random intercepts for participants in the model to mimic the repeated measures ANOVA. The results regarding ratings of change in social distance were in line with the findings reported above, showing a significant main effect of secrecy, *b* = 0.27, *SE b* = 0.12, *t*(243.96) = 2.38, *p* = .018, and of valence, *b* = 0.67, *SE b* = 0.12, *t*(243.96) = 5.80, *p* < .001, but no significant interaction, *b* = -0.11, *SE b* = 0.23, *t*(243.96) = -0.46, *p* = .645. When looking at the ratings on the IOS scale, we found a significant main effect of valence, *b* = -0.68, *SE b* = 0.15, *t*(259.22) = -4.65, *p* < .001, whereas the main effect of secrecy did not reach significance, *b* = -0.25, *SE b* = 0.15, *t*(259.22) = -1.71, *p* = .089. The interaction was not significant either, *b* = .05, *SE b* = 0.29, *t*(259.22) = 0.18, *p* = .861. Including random intercepts for the scenarios did not change the resulting fixed effects.

### Discussion

Study 1 investigated whether participants perceived the social distance between themselves and another person as smaller when the exchanged information was secret compared to when it was not secret, and whether this change differed depending on the valence of the content. The results we obtained provide mixed support for our hypotheses.

When asking about perceptions of change in social distance, we found that exchanging secret information led to ratings of more closeness compared to exchanging the same information not labeled as secret. This was true for the direct assessment of change in distance, providing support for Hypothesis 1. The same pattern was found descriptively when using a pictorial measure of closeness, the IOS scale, however, this difference was not significant. We also found that valence impacted the change ratings, showing that positive content resulted in more closeness than negative content. This was qualified by an interaction with scenario, indicating that this pattern was present for some but not all scenarios. However, it is important to note that the interaction effect is difficult to interpret, as the content of the audio stories was randomly allocated to the scenario number. This means that, for example, in the first scenario a job offer (positive valence condition) is compared with an addiction (negative valence condition). Content is therefore not constant between valence conditions. In an exploratory fashion, however, we looked at the descriptive results for each scenario. In scenarios 2, 3, and 5, positive information resulted in more closeness than negative information, whereas the pattern was opposite for scenarios 1 and 4. While all positive scenarios describe a happy incident, the negative scenarios might critically differ: Scenario 1 concerns an addiction and scenario 4 a betrayal and a desire for divorce. While both are negative, the stories are framed more as something that happened to the sharer (the sharer could be identified as a victim) and they also reveal a high level of vulnerability and potentially trust in the receiver; see also [55]. Scenarios 2 (starting rumors), 3 (hating the supervisor / thinking about quitting one’s job), and 5 (stealing an idea) contain a different element, they are more active (the sharer may be identified as a transgressor) and potentially leave the sharer in a less vulnerable position. Perceptions of agency (or more specifically transgression) and vulnerability might therefore be the element that determines whether sharing negative content may reduce social distance to a larger or smaller extent.

When interpreting the effects of secrecy (H1), we further need to highlight a finding in regards to our manipulation: Participants rated the content in the secret condition as more confidential than in the information condition. However, when exploratorily looking at confidentiality not only across secret but also across valence conditions, we found that negative information was rated as almost as confidential as the negative and positive secrets, *M*_*secret-neg*_ = 5.94 (*SD* = 1.43), *M*_*secret-pos*_ = 5.90 (*SD* = 1.23), *M*_*info-neg*_ = 5.78 (*SD* = 1.52), *M*_*info-pos*_ = 4.78 (*SD* = 1.77). It appears that sharing negative information may be perceived similarly to sharing a secret, no matter whether it is qualified by a respective tag (e.g., “please don’t tell anyone”, see Materials) or not. These results are in line with findings from Pederson and McLaren [56], who investigated the setting of explicit and implicit privacy rules. They show that even in the absence of explicit privacy rules (e.g., “please don’t tell anyone”), people might perceive information as secretive and they could even anticipate implicit privacy rules, especially when it comes to negative information. For instance, negative information may be perceived as more confidential because receivers may be reluctant to disclose this information if it were about themselves. Negative information, therefore, might have been implicitly understood as confidential, and whether it was labelled as such possibly did not matter. This might explain why the effect of secrecy did not always reach significance across all dependent variables and analyses, as the difference in confidentiality between secret and not secret messages was only perceived for the positive scenarios and not so much for the negative scenarios.

While our data provide partial support for Hypothesis 1 (sharing secrets reduces social distance more than sharing information when looking at the direct assessment, whereas the effect does not reach significance for pictorial assessments), we do not find support for Hypothesis 2 (an interaction between secrecy and valence). Analyses with alternative, not preregistered methods such as robust analyses or linear mixed models further confirm these findings. Our results thereby show that exchanging secret information may lead to perceptions of more closeness than when exchanging non-secret information, and that on average exchanging positive content leads to more closeness than when exchanging negative content–however, this depends on the pairing of specific scenarios (see above).

When investigating individuals’ personal experiences instead of providing them with fictitious examples, sharing positive information also results in more closeness. Yet, the secrecy effect disappears, meaning that whether one shares a secret or other information does not affect perceived closeness. These data, however, also contain more noise because of the many different situations participants described.

## Study 2

Study 1 tested our idea that sharing secrets decreases social distance with a focus on the receiver. In Study 2, we aimed at learning about changes in distance from an observer’s point of view and used a different methodological approach, namely vignettes. As is the case with recordings but not with participants’ experiences, written vignettes allow for describing a concrete situation while keeping both the content of the information exchange as well as previous levels of closeness constant. This provided us with a promising approach to study intimate relationships [57] and contributes to the internal validity of our study. To further explore the potential role of how identity-relevant the shared information was (and how this might impact ratings of change in closeness), we asked participants to rate this aspect for each vignette at the end of the study.

Applying our hypotheses to this context, we expected that learning that two persons have exchanged a secret (rather than information only) results in lower perceived social distance between these individuals (Hypothesis 1). Furthermore, Study 2 again investigated whether changes in social distance depend on the nature of the information shared, meaning whether it was positive or negative (Hypothesis 2).

### Methods

#### Participants

The study was conducted as an online study advertised as a *study on the evaluation of behavior* via Prolific. Our a priori power analysis with an α-level of .05, a desired power of .95, and a small to medium effect size estimate (*f* = 0.15) while assuming a small correlation between repeated measures (.20) indicated a required sample size of 280 participants [47, 48]. We increased this number by 10% (while ensuring that cell numbers were balanced) to reach the required sample size even if participants need to be excluded due to prescreening and the exclusion criteria described below. The aspired sample therefore comprised 308 participants.

As a prescreening criterion, we required all participants to speak English as their native language. Furthermore, participants who had previously worked on Study 1 were not eligible to participate in Study 2. Participants who did not give consent were screened out from the survey. Additionally, eligible participants were asked to indicate whether they saw any reason as to why their data should not be used for statistical analyses at the end of the study. If they asked for exclusion, we did not use their data for our analyses. We analyzed complete data sets only.

Three hundred sixteen participants started the study of which 310 provided written informed consent, completed the study, and received £0.87 as compensation. One participant indicated being confused as a reason to exclude their data, which we accordingly did. Even though we set English as the native language as a prescreening criterion on Prolific, 25 participants indicated that they spoke English fluently but that it was not their native tongue, and three participants only had a basic knowledge of English. We excluded these participants from our analyses as well, which resulted in a final sample of 281 participants (152 female, 128 male, one undisclosed, *M*_*age*_ = 35.15, *SD*_*age*_ = 12.42). Participants were randomly assigned to four between-subjects conditions with 72 participants reading positive secrets, 67 negative secrets, 70 messages with positive information, and 72 messages with negative information.

#### Design

Study 2 was set up as a mixed design with two between-subjects factors (secrecy: message framed as secret vs. not, and valence: information is positive vs. negative) and one within-subjects factor (vignette number: one to five). Perceived change in social distance between the interaction partners described in the vignettes served as the dependent variable and was assessed directly with a Likert scale and a pictorial measure.

#### Materials

The complete set of vignettes with positive content can be found in the S1 File and the set of vignettes with negative content can be found in the S2 File. The instructions and questions within the study were similar to those of Study 1, see below.

#### Procedure

The procedure of Study 2 was identical to Study 1, except that participants were asked to evaluate the anticipated change between the persons described in the vignettes (“After having read the interaction between [names of interaction partners], do you perceive that their relationship might have changed to some extent?”; answer options ranging from 1 = *not at all* to 7 = *very much*; and in case they indicated a change “Do you perceive the relationship between [names of interaction partners] as closer or more distant than before?”; 1 = *closer than before*; 7 = *more distant than before*).

In order to investigate potential differences between the content of the information shared (meaning the vignettes) in an exploratory fashion, we further asked participants to reread the vignettes at the end of the study and rate how relevant they thought the information was to the identity of the secret-sharer (1 = *very irrelevant*, 7 = *very relevant*).

### Results

#### Preregistered data analysis: Confidentiality of information

The data analysis procedure was the same as in Study 1. After excluding all participants according to our a priori defined criteria (see above), we used a repeated measures ANOVA to analyze whether participants perceived vignettes framed as secret compared to vignettes not framed as secret as more confidential. This analysis served as a check to test whether our manipulation of secrecy was successful. Looking at means averaged across scenarios, the scenarios were generally rated as rather confidential, but more so in the secrets conditions compared to the not secret condition; *M* = 5.58, *SD* = 0.91 versus *M* = 4.81, *SD* = 1.16, *F*(1, 279) = 38.11, *p* < .001, η_p_^2^ = .12. We further found a significant main effect of scenario, *F*(3.15, 878.19) = 6.88, *p* < .001, η_p_^2^ = .02, indicating that confidentiality ratings differed between scenarios, which was not qualified by a secrecy-scenario interaction, *F*(3.15, 878.19) = 2.31, *p* = .072, η_p_^2^ = .01 (see Table 3, but note that ratings per scenario were collapsed across valence conditions, meaning that different information is grouped together, which is why we did not further analyze scenario effects). As several assumptions of the ANOVA were violated, we also used the WRS2 package [50] to compute robust tests (not preregistered). As with the ANOVA, there were significant main effects of secrecy (*Q*(1, 160.84) = 32.51, *p* < .001) and scenario (*Q*(4, 132.76) = 11.96, *p* < .001). Unlike the ANOVA results, we also found a significant interaction, *Q*(4, 132.76) = 4.85, *p* = .001 (but see issues regarding its interpretation above).

**Table 3:** Overview of means (and standard deviations) from Study 2 without listwise deletion (see ANOVA analysis above).

Dependent variable	Condition		Scenario					
	Secrecy	Valence	1	2	3	4	5	Average
Confidentiality	Secret	Collapsed	5.74 (1.41)	5.42 (1.43)	5.63 (1.26)	5.60 (1.56)	5.50 (1.57)	5.58 (0.91)
	Non-secret	Collapsed	5.34 (1.74)	4.56 (1.78)	4.85 (1.41)	4.53 (2.09)	4.80 (1.58)	4.81 (1.16)
Extent of change	Secret	Positive	3.54 (1.68)	3.14 (1.59)	4.00 (1.64)	2.90 (1.64)	3.68 (1.86)	3.45 (1.29)
		Negative	4.87 (1.44)	5.37 (1.48)	3.15 (1.58)	3.69 (1.91)	5.18 (1.13)	4.45 (0.89)
	Non-secret	Positive	4.13 (1.40)	3.26 (1.63)	3.74 (1.64)	3.06 (1.64)	3.73 (1.70)	3.58 (1.16)
		Negative	4.96 (1.40)	5.61 (1.13)	3.58 (1.55)	3.92 (1.74)	5.18 (1.36)	4.65 (0.84)
Change in social distance	Secret	Positive	3.76 (1.37)	3.11 (1.16)	3.44 (1.20)	3.49 (1.12)	3.04 (1.55)	3.40 (0.90)
		Negative	3.04 (1.36)	5.09 (1.53)	3.46 (1.11)	2.72 (1.56)	5.06 (1.00)	3.96 (0.77)
	Non-secret	Positive	4.16 (1.24)	3.60 (1.27)	3.48 (1.13)	3.78 (1.08)	3.15 (1.51)	3.66 (0.82)
		Negative	3.16 (1.40)	4.92 (1.49)	3.76 (1.24)	2.81 (1.52)	5.20 (1.12)	4.03 (0.74)
IOS scale	Secret	Positive	5.24 (1.06)	6.00 (0.94)	5.78 (0.85)	5.62 (1.23)	6.13 (1.21)	5.77 (0.78)
		Negative	5.97 (1.38)	4.30 (1.98)	5.13 (1.21)	6.35 (1.20)	4.09 (1.65)	5.13 (0.98)
	Non-secret	Positive	4.73 (1.61)	5.88 (1.27)	5.26 (1.37)	5.15 (1.41)	6.03 (1.17)	5.40 (0.94)
		Negative	5.84 (1.36)	4.13 (2.02)	4.94 (1.50)	6.38 (1.07)	4.04 (1.70)	5.01 (1.16)
Relevance to identity	Secret	Positive	5.50 (1.23)	5.54 (1.37)	5.58 (1.43)	5.64 (1.26)	5.81 (1.40)	5.61 (0.94)
		Negative	6.27 (0.88)	5.46 (1.28)	4.46 (1.73)	5.91 (1.29)	5.30 (1.52)	5.48 (0.81)
	Non-secret	Positive	5.26 (1.37)	5.71 (1.19)	5.44 (1.55)	5.37 (1.41)	5.96 (1.17)	5.55 (0.92)
		Negative	5.69 (1.38)	5.76 (1.22)	4.51 (1.63)	5.54 (1.45)	5.26 (1.50)	5.36 (0.89)
Additional variables	Secrecy	Valence	1	2	3	4	5	Average
Length of conversation	Secret	Positive	3.52 (1.17)	3.75 (1.23)	3.51 (1.19)	3.43 (1.10)	4.04 (1.28)	3.60 (0.96)
		Negative	4.78 (1.27)	3.95 (1.31)	3.93 (1.18)	5.20 (1.25)	2.91 (1.08)	4.09 (0.91)
	Non-secret	Positive	3.79 (1.31)	3.75 (1.29)	3.81 (1.45)	3.47 (1.32)	4.08 (1.43)	3.73 (1.03)
		Negative	4.49 (1.51)	3.69 (1.35)	3.51 (1.13)	5.00 (1.32)	2.96 (1.13)	3.89 (0.97)
Expected next meeting	Secret	Positive	3.45 (1.29)	3.04 (1.32)	3.24 (1.10)	3.42 (1.15)	3.34 (1.48)	3.24 (0.78)
		Negative	2.99 (1.35)	4.00 (1.50)	3.46 (0.97)	2.54 (1.25)	4.01 (1.40)	3.41 (0.79)
	Non-secret	Positive	3.99 (1.59)	3.12 (1.44)	3.98 (1.36)	3.75 (1.21)	3.18 (1.35)	3.64 (0.89)
		Negative	3.09 (1.35)	4.22 (1.33)	3.67 (1.22)	2.84 (1.32)	4.48 (1.12)	3.71 (0.71)
Location of next encounter	Secret	Positive	3.60 (1.24)	4.54 (1.28)	4.35 (0.95)	4.02 (0.93)	4.57 (1.17)	4.23 (0.59)
		Negative	4.99 (1.50)	4.02 (1.61)	4.20 (1.28)	5.11 (1.25)	3.69 (1.52)	4.41 (0.84)
	Non-secret	Positive	3.34 (1.38)	4.18 (1.28)	4.27 (1.20)	4.02 (1.21)	4.54 (1.27)	4.03 (0.83)
		Negative	5.20 (1.30)	4.14 (1.60)	4.03 (1.32)	5.37 (1.15)	3.59 (1.44)	4.43 (0.68)

### Preregistered data analysis: Change in social distance (direct assessment)

**Fig 2:**
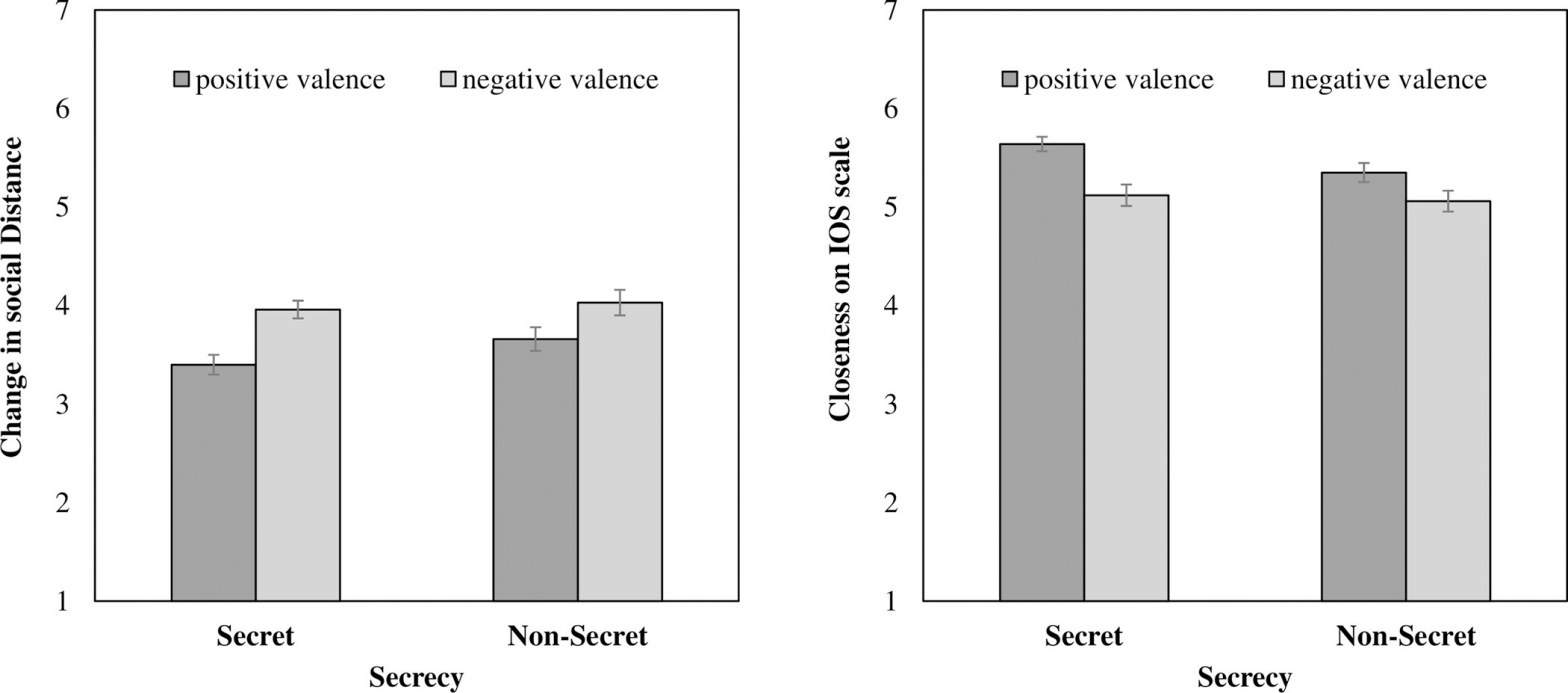
Effects of secrecy and valence on change in social distance (left) and on closeness on the IOS scale (right) in Study 2. Change in social distance and IOS closeness ratings range from 1 (closer than before / largest distance between circles) to 7 (more distant than before / largest overlap between circles). Error bars represent standard errors.

With repeated measures ANOVAs, we analyzed whether participants perceived the change in social distance as different depending on whether the exchanges were framed as secrets or not (main effect of secrecy). Furthermore, we tested whether the change in social distance depended on the valence of the content (interaction effect between secrecy and valence). We calculated this analysis once for the direct evaluation of social distance and once for the IOS scale measure (for all recordings in which participants indicated that a change had occurred). As the repeated measures ANOVA applies listwise deletion (see Study 1), the repeated measures ANOVA reduced the sample to 193 (69% of the sample). Results for the direct evaluation of change in social distance indicated a non-significant main effect of secrecy, *F*(1, 189) = 3.76, *p* = .054, η_p_^2^ = .02. Descriptively, all participants thought that the two individuals would feel closer to each other than before (means < 4), but, non-significantly, more so in the condition where the information was framed as secret versus not framed as secret, *M* = 3.61, *SD* = 0.77 versus *M* = 3.81, *SD* = 0.72, see Fig 2. Results again yielded an unexpected significant main effect of valence, *F*(1, 189) = 15.14, *p* < .001, η_p_^2^ = .07, indicating that distance decreased more in the positive compared to the negative valence condition, *M* = 3.50, *SD* = 0.77 versus *M* = 3.90, *SD* = 0.67. We again found a significant main effect of scenario, *F*(3.81, 719.35) = 22.80, *p* < .001, η_p_^2^ = .11, indicating that distance decreased more or less depending on the scenario (again collapsed across valence conditions and therefore not further analyzed). The main effect of valence was qualified by a significant interaction between valence and scenario, *F*(3.81, 719.35) = 58.78, *p* < .001, η_p_^2^ = .24. The descriptive results for scenarios 2, 3, and 5 indicate that distance decreased more in the positive compared to negative condition, whereas in scenarios 1 and 4 the opposite pattern occurred. We did not find the hypothesized interaction between secrecy and valence, *F*(1, 189) = 1.98, *p* = .161, η_p_^2^ = .01, or other interaction effects, all *F*s < 0.74. As several assumptions of the ANOVA were violated, we used the WRS2 package [50] to compute a robust test for the between-condition effects (not preregistered). Different to the ANOVA, there was a significant main effect of secrecy (*Q* = 4.21, *p* = .043), a significant main effect of valence (*Q* = 19.64, *p* = .001), and no significant interaction between secrecy and valence (*Q* = 3.29, *p* = .073).

#### Preregistered data analysis: Change in social distance (pictorial assessment)

When analyzing the data regarding change in social distance assessed with the pictorial measure, we noticed a programming error. Participants were asked to evaluate social distance even if they had indicated no change. To correct for this, we created a new variable for each scenario that only contained values for the trials in which participants had indicated that they thought a change in the relationship had occurred.

The perception of distance assessed with the IOS-scale revealed similar patterns. Results from a repeated measures ANOVA indicated a non-significant effect of secrecy, *F*(1, 189) = 1.75, *p* = .188, η_p_^2^ = .01. Descriptively, participants indicated more closeness (more overlap between the circles) in the secret compared to not secret condition, *M* = 5.37, *SD* = 0.88 versus *M* = 5.19, *SD* = 1.04, yet this difference was non-significant (see above). We again found a non-hypothesized main effect of valence, *F*(1, 189) = 8.71, *p* = .004, η_p_^2^ = .04, indicating more closeness in the positive compared to the negative valence condition, *M* = 5.49, *SD* = 0.87 versus *M* = 5.09, *SD* = 1.02. The analysis also replicated the effect of scenarios, *F*(3.77, 712.53) = 17.60, *p* < .001, η_p_^2^ = .09, which was qualified by an interaction between scenarios and valence, *F*(3.77, 712.53) = 72.86, *p* < .001, η_p_^2^ = .28. Descriptive results again indicate that for scenarios 2, 3, and 5, closeness was rated more highly in the positive compared to the negative condition, whereas the opposite pattern occurred in scenarios 1 and 4. Different from our hypothesis, the interaction between secrecy and valence was not significant, *F*(1, 189) = 0.66, *p* = .417, η_p_^2^ = .00, which was also true for the remaining interaction effects, all *F*s < 0.90. Computing a robust analysis [50] in regards to the between-conditions effects (not preregistered) corroborated the ANOVA: There was no significant main effect of secrecy (*Q* = 0.98, *p* = .325), a significant main effect of valence (*Q* = 6.54, *p* = .012), and no significant secrecy-valence interaction (*Q* = 0.63, *p* = .429).

#### Preregistered data analysis: Identity relevance as a covariate

As an exploratory analysis, we further included the judgments on the relevance of the information shared regarding the identity of the sharer (see Table 3 for descriptive results). We added relevance as an additional factor (z-standardized) and analyzed the data using linear mixed models [51] using the lme4 and lmerTest package [52, 53]. Using the package rstatix [54], we excluded extreme outliers before computing the analysis (3 data points for social distance and 6 data points for the IOS scale) and used effect coding for secrecy and valence (-0.5 for *secrets*, 0.5 for *information*; -0.5 for *positive valence*, 0.5 for *negative valence*). We included random intercepts for participants in the model. The results regarding ratings of change in social distance were mostly in line with the findings reported above, showing a non-significant main effect of secrecy, *b* = 0.18, *SE b* = 0.09, *t*(255.91) = 1.89, *p* = .060, a significant main effect of valence, *b* = 0.45, *SE b* = 0.09, *t*(255.91) = 4.89, *p* < .001, but no significant interaction, *b* = -0.19, *SE b* = 0.19, *t*(255.91) = -1.03, *p* = .305. Including random intercepts for scenario did not change the resulting pattern.

When looking at the ratings on the IOS scale, we also found a significant main effect of valence, *b* = -0.53, *SE b* = 0.12, *t*(265.42) = -4.55, *p* < .001. The main effect of secrecy did not reach significance, *b* = -0.22, *SE b* = 0.12, *t*(265.42) = -1.93, *p* = .054. The valence-secrecy interaction was not significant, *b* = 0.24, *SE b* = 0.23, *t*(265.42) = 1.05, *p* = .295. When including a random intercept for scenario, the main effect of secrets reached significance; *b* = -0.23, *SE b* = 0.12, *t*(265.93) = -1.99, *p* = .048.

Including relevance did not change the significance levels of the results: While relevance had a significant effect on both the direct assessment of closeness, *b* = -0.10, *SE b* = 0.04, *t*(1140.96) = -2.20, *p* = .028, and on the closeness ratings using the IOS scale, *b* = 0.20, *SE b* = 0.05, *t*(1222.85) = 4.43, *p* < .001, previously (not) significant main effects remained (not) significant, and the secrecy-valence interaction remained not significant.

#### Preregistered data analysis: Identification of the research question

We also analyzed how many participants had correctly guessed our research question and whether excluding them affected our results. We again asked a research assistant to code the open answers. When asked, 72 participants (i.e., 26%) thought that the study was related to secrets and/or interpersonal relations (however, this might simply reflect that they paid attention to the instructions throughout the study). Excluding this part of our sample in addition to participants who had missing values on the main dependent variables (due to listwise deletion) resulted in a sample of 145 participants. When rerunning the repeated measures ANOVAs, we found the same pattern of results regarding perceptions of distance (directly assessed and measured via the IOS scale): We found no significant main effect of secrecy, whereas the main effects of valence and scenario remained significant and were qualified by the previously found interaction effect between valence and scenario. Using robust analyses [58] resulted in the same findings.

### Discussion

The results from Study 2 provide weak support for our hypotheses, and partly replicate the patterns found in Study 1. As hypothesized, participants rated the content in the secret condition as more confidential than in the information condition.

Regarding perceptions of change in social distance, we only found non-significant descriptive tendencies that sharing secrets (compared to sharing information) changed to ratings of closeness with the preregistered analyses. This was true for the direct assessment of change in distance as well as when using a pictorial measure of closeness, the IOS scale. Looking at the results with mixed models, the main effect of secrecy was only significant for the pictorial assessment of closeness (showing that secrecy was associated with more closeness) but not for the direct assessment. Taken together, we only find weak support for Hypothesis 1 in this study.

We also found that valence impacted the change ratings, showing that positive content resulted in more closeness than negative content. This was qualified by an interaction with scenario, indicating that this pattern was only observed for some scenario combinations (2, 3, and 5), whereas for others the opposite pattern occurred (1 and 4). Interestingly, this pattern replicated the findings from Study 1 and indicated that the topics covered in the vignettes might critically influence the reactions (see S1 File and S2 File). When looking at descriptive results (Table 3) in an exploratory fashion, we again see that positive secrets appear to lead to a decrease in distance and more closeness, while effects vary more for the negative vignettes. We again speculate that this is related to the nature of the vignettes’ content. Revealing something negative and showing vulnerability (e.g., being betrayed and wanting a divorce) could lead to a different change in perceived distance than a story where a person is a transgressor and did something negative by harming others (e.g., spreading rumors).

When exploratorily looking at confidentiality not only across secret but also across valence conditions, we found that negative information was rated as almost as confidential as the negative and positive secrets, *M*_*secret-neg*_ = 5.66 (*SD* = 0.87), *M*_*secret-pos*_ = 5.51 (*SD* = 0.94), *M*_*info-neg*_ = 5.44 (*SD* = 0.89), *M*_*info-pos*_ = 4.17 (*SD* = 1.05). This could again indicate that sharing negative information may be perceived as very similar to sharing a secret, whether it is qualified by an explicit request to keep it to oneself or not. Again, participants could anticipate the existence of implicit privacy rules [56], especially when the vignette contains negative content. This, in turn, could explain why effects of secrecy do not reach significance–the between-subjects manipulation was possibly not as strong as we had hoped for, explaining why effects found are rather small. It might further be that, in general, written vignettes are less engaging for participants than audio recordings and therefore lead to smaller effects. Similarly, thinking about how a relationship between two hypothetical individuals might change is likely less engaging than when participants are asked to imagine being the individual affected by the information exchange, for example, because participants might have been in a similar situation before and were therefore more involved.

In line with Study 1, we did not find any support for Hypothesis 2, meaning an interaction between secrecy and valence. Again, this could be due to a not ideal manipulation of secrecy. Instead, we replicated the previously found main effect of valence, showing that participants indicated higher levels of closeness after reading about the sharing of positive versus negative information. This main effect was, however, qualified by an interaction, showing that effects might depend on what information is presented in the negative and positive condition and contrasted against each other (an addiction versus a job offer in scenario 1 or a marriage proposal versus a rumor that harmed another person in scenario 2).

When including participant ratings of the shared content’s relevance using mixed models, we found that relevance positively predicted closeness: When the content was more relevant to the sharer’s identity, participants rated the relationship between the interaction partners as closer than before. We did not anticipate this effect, but it is in line with our assumption that sharing secrets, which are presumably more relevant to the self (compared to information), could signal trust or the need for support [11] and therefore positively influence relationships between individuals. Similarly, because trust is a common reason for disclosing personal information in close relationships [59], a person may be perceived as more likely to share a secret relevant to their identity with someone they feel close to.

## Study 3

While Study 1 focused on the perspective of the receiver and Study 2 on the perspective of an observer, Study 3 investigated the sharing of secrets from the sharer’s point of view in a lab setting where individuals were asked to either create social closeness or social distance by eventually sharing secrets. Study 3 thereby investigated whether participants’ choices and behavior regarding information sharing differed depending on the goal they had when interacting with others. We hypothesized that individuals were more likely to share secrets (vs. information) when they wanted to create social closeness compared to distance (Hypothesis 1), but that they would choose to share more or fewer negative secrets in one of the two conditions (Hypothesis 2).

### Methods

#### Participants

The study was conducted in the lab and advertised as *study on information sharing* via different university platforms. Data collection took place during the COVID-19 pandemic.

Our a priori power analysis with an α-level of .05, a desired power of .80, and a medium effect size estimate for the within-between interaction term (*f* = 0.25) indicated a required sample size of 128 participants [47, 48]. We increased this number by 10% (while ensuring that cell numbers were balanced) to reach the required sample size even if participants needed to be excluded due to the criteria described below. The aspired sample would therefore ideally comprise 142 participants. We had preregistered that we would collect data across a minimum time span of two months to ensure that we reached the sample size we aimed for. Due to COVID-related restrictions at the university, data collection took place across one year with several longer breaks in between due to lockdowns. We analyzed the data only after data collection was completed.

One hundred forty-four participants started the study and 141 completed it. All participants gave written informed consent. Additionally, eligible participants were asked to indicate whether they saw any reason as to why their data should not be used for statistical analyses at the end of the study. Six participants self-reported reasons or concerns and were therefore excluded from our analyses. The remaining sample consists of 135 participants (39 male, 94 female, 2 no information; *M*_*age*_ = 24.19, *SD* = 5.11). Sixty-seven participants were randomly assigned to the closeness condition and 68 to the distance condition.

#### Design

Study 3 was set up as a mixed design with one between-subjects factor (goal: closeness vs. distance) and one within-subjects factor (type of information: general information vs. positive secrets vs. negative secrets). Amount of shared information (zero to three) served as a dependent variable.

#### Materials and procedure

First, participants were asked to think about their own lives and to deliberate on general information that described themselves, but also on secrets that they might have and would not feel comfortable to just share with anybody. They were next asked to narrow down these thoughts to three pieces of general information about themselves, three positive secrets, and three negative secrets. Importantly, participants were not asked to write down these secrets, but to just think of them and provide a keyword so they could later recognize them.

Second, participants learned that we would like to understand how they behave in a typical university context: a first day in class. They should imagine joining a new class in which they did not know anyone yet. We then asked participants how they would act when starting to work with another student in the class. They were either told that they would continuously work with this person and therefore aim to create a feeling of closeness (closeness condition) or that they would probably not work with this student again and therefore were not interested in creating a feeling of closeness (distance condition).

Third, we asked them which information they would share with the other student. Participants then saw the nine keywords they had previously entered and were asked to indicate which of the pieces of information they would share. Participants were asked to choose three pieces of information–a number that allowed them to share only information, only positive secrets, only negative secrets, or any combination of those three types of information.

After collecting the data on sharing behavior, we asked participants to complete a manipulation check. We again showed participants their keywords and asked how difficult they would find it to talk about this aspect with a stranger (manipulation check for secrecy; 1 = *not difficult at all* to 7 = *very difficult*) and how positive or negative this piece of information was (manipulation check for valence; 1 = *very negative* to 7 = *very positive*).

Finally, participants were asked to provide demographic information (gender and age) and whether they saw any reasons as to why their data should not be included in the study. At the end of the study, individuals were debriefed and thanked for their participation.

### Results

#### Preregistered data analysis

All participants provided nine pieces of information about themselves (using a keyword only). The manipulation check showed that participants rated neutral information as less secret (*M* = 1.60, *SD* = 0.80) than the positive (*M* = 4.22, *SD* = 1.76) and negative secrets (*M* = 5.89, *SD* = 1.63). Looking at valence, both the neutral information (*M* = 5.61, *SD* = 0.98) and positive secrets were rated as positive (*M* = 5.49, *SD* = 0.89), and negative secrets were rated as negative (*M* = 2.62, *SD* = 0.77).

Participants were asked to share three pieces of information in the study. On average, participants shared 2.10 neutral pieces of information (*SD* = 0.83) and 0.90 secrets (*SD* = 0.83). Looking at the secrets, participants on average shared more positive secrets (*M* = 0.60, *SD* = 0.70) than negative secrets (*M* = 0.30, *SD* = 0.61) with the other student. This pattern was present in the distance condition (neutral information: *M* = 2.22, *SD* = 0.90; positive secrets: *M* = 0.49, *SD* = 0.66; negative secrets: *M* = 0.29, *SD* = 0.69) and in the closeness condition (neutral information: *M* = 1.99, *SD* = 0.75; positive secrets: *M* = 0.72, *SD* = 0.73; negative secrets: *M* = 0.30, *SD* = 0.52).

To test our hypotheses, we preregistered two repeated measures ANOVAs to analyze a) whether participants shared more secrets in relation to general information in the closeness condition (interaction effect of goal and type of information, i.e., general information vs. secret), and b) whether the proportion of positive to negative secrets was different in the closeness and in the distance condition (interaction effect of goal and valence of secret).

For the first analysis, we added up the positive and negative secrets and counted the average number of shared secrets compared to general information shared to obtain our dependent variable. Results from the ANOVA analysis indicate a non-significant effect of goal, *F*(1, 133) = -0.00, *p* = 1.00, η_p_^2^ = 0.00, and a significant main effect of type of information, *F*(1, 133) = 71.96, *p* < .001, η_p_^2^ = 0.35. The main effects were not qualified by a significant interaction, *F*(1, 133) = 2.75, *p* = .100, η_p_^2^ = 0.02. Participants were generally more likely to share neutral information compared to secrets (see descriptive results above), and this tendency did not differ significantly between participants with closeness or distance goals.

For the second analysis, we compared the number of positive and negative secrets shared. Results from the ANOVA analysis indicated a non-significant effect of goal, *F*(1, 133) = 2.75, *p* = 0.10, η_p_^2^ = 0.02, and a significant main effect of valence of the secret, *F*(1, 133) = 12.00, *p* < .001, η_p_^2^ = 0.08. The main effects were not qualified by a significant interaction, *F*(1, 133) = 1.66, *p* = .200, η_p_^2^ = 0.01. Participants were generally more likely to share positive secrets compared to negative secrets (see descriptive results above), and this tendency did not differ significantly between participants with closeness or distance goals.

In addition to the preregistered analyses, we further tested the hypothesis using mixed models to take into account that our dependent variable was count data. Each of the nine pieces of information were modelled as one trial, and selection of the piece of information (1 = *selected*, 0 = *not selected*) served as a binary outcome variable. Participants’ goal and type of information / valence of secret were effect-coded using -0.5 (*closeness / information / positive*) and 0.5 (*distance / secret / negative*). The results from the mixed model analyses corroborate the analyses above: We found significant effects of type of information (*b* = -3.19, *SE b* = 0.34, *z* = -9.33, *p* < .001) and valence of the secrets (*b* = -0.89, *SE b* = 0.27, *z* = -3.26, *p* = .001), but no effect of goal (*b* = 0.02, *SE b* = 0.16, *z* = 0.14, *p* = .891; *b* = -0.30, *SE b* = 0.23, *z* = -1.33, *p* = .182) and no interaction effect (*b* = -0.97, *SE b* = 0.50, *z* = -1.95, *p* = .051; *b* = 0.52, *SE b* = 0.54, *z* = 0.95, *p* = .340).

### Discussion

Our results indicate that participants were more likely to share neutral information than secrets. If they decided to share a secret, it was more likely a positive than a negative secret. This pattern did not significantly differ for participants who had the goal to create closeness and who were presumably not interested in creating closeness between themselves and the other student.

It could be that participants were not particularly willing to share secrets with a stranger. As we outlined in the introduction, although previous research indicates that people can be willing to share personal information with “a passing stranger” [60], people are presumably more willing to confide secrets in closer others with certain attributes [12]. In other words, a certain level of acquaintance might be required before people even consider (strategically) sharing secrets and the sharing of especially negative pieces of information further needs to be perceived as appropriate in the respective situation to not result in avoidance [55]. Participants, furthermore, may not anticipate the positive effects that sharing a (positive) secret may have on the development of a relationship. Alternatively or additionally, the goal manipulation might not have been ideal to change sharing behavior. The situation could have been perceived as artificial and participants might have perceived it to be unnecessary to share secretive information to work amicably with another student. If participants, furthermore, expected to work together with a student in the future anyway, they might have thought that their relationship would become closer over time, without them needing to reveal personal secrets.

Interestingly, participants rated both neutral information and positive secrets as more positive compared to negative secrets. This further emphasizes that, irrespective of expected future interactions, especially positive pieces of information (including secrets) were shared with others. Potentially, this points at an implicit tendency to create closeness with others, for instance, to satisfy individuals’ need to belong [61]. Alternatively, when interacting with a person one will likely not form a close bond with, engaging in a conversation with minimal self-disclosure, e.g., small-talk [62], may be a more common strategy than disclosing neutral information.

In order to tap into strategic sharing behavior, future work could use more realistic scenarios where more information is provided about the receiver, where sharing follows an acquaintance period, and/or where the goal of creating closeness or distance at the expense of sharing personal secretive information is more strongly incentivized. All in all, however, Study 3 aligns with previous research showing that individuals can be hesitant to share even small secrets, eventually as they overestimate the receiver’s negative reaction to their self-disclosure of fears or vulnerabilities [24, 25].

## General discussion

In this article, we investigate how sharing (secret) information of positive and negative valence impacts perceived change in social distance between the sharer and receiver. We investigate this using online (Studies 1 and 2) and lab studies (Study 3) and a variety of materials such as auditory files (Study 1), vignettes (Study 2), and a behavioral setting (Study 3) to gain a better understanding of the relational consequences of sharing secrets. Throughout all studies, we tested two hypotheses, namely that (1) sharing secrets compared to non-secret information decreases perceived distance and (2) this association differs depending on the valence of the secrets.

Study 1 investigated the *receiver’s* perspective on the information exchange by asking participants to listen to auditory recordings of people sharing (secret) negative versus positive information and to recall a personal experience where somebody shared (secret) negative versus positive information with them. Results provided support for Hypothesis 1, showing that exchanging secret (vs. non-secret) information resulted in more closeness than before the exchange when assessing change in distance directly. Furthermore, when sharing positive compared to negative content, participants rated social distance as lower. This effect was not consistent across scenario pairings, but was also not qualified by secrecy (i.e., whether the content was labeled as secret or not). Results therefore do not provide support for Hypothesis 2.

Study 2 investigated the *observer’s* perspective by asking participants to read five vignettes in which one person shared secret or non-secret information of positive versus negative valence with someone else. Descriptively, observers rated sharing secrets as leading to more closeness compared to information. This descriptive difference, however, was not significant. When investigating the pattern with mixed model analyses, these differences were partly significant; however, the use of mixed model analyses was not preregistered. Sharing positive compared to negative content resulted in more closeness, yet this pattern again differed across scenarios. Results did not provide support for Hypothesis 2, as we found no significant interaction between valence and secrecy on closeness ratings. Study 2 further showed that identity-relevant content positively predicts closeness, meaning that the more participants perceived the information to be relevant to the sharer’s identity, the closer they thought the sharer and recipient would be after the information exchange.

Study 3 aimed to tap into the role of the *sharer* and investigate the potentially strategic use of sharing secrets to increase or decrease distance from someone else. Results show that participants were much more willing to share general information than secrets. If they did decide to share a secret, it was more likely a positive than a negative one. This pattern did not significantly differ between the conditions where participants aimed to create versus not create closeness with the other student.

All in all, results partly support the hypothesis that sharing secrets changes social distance and increases closeness. Sharing positive compared to negative information can furthermore increase closeness between sharer and receiver—at least from the receiver’s (Study 1) or from the observer’s (Study 2) point of view. This is in line with current research showing that sharing (and not hiding) successes increases closeness [63]. Studies 1 and 2 also indicate that, in some cases, sharing negative information might increase closeness, too. When looking at the respective vignettes in an exploratory fashion, it appears that scenarios describing an addiction (Scenario 1) and a betrayal and desire for a divorce (Scenario 4) result in more closeness despite them being negative. Both scenarios, however, might be framed as something negative that happened to the secret sharer and could be associated with fear of discovery, see also [64]. By sharing this secret, the sharer may show high levels of vulnerability and trust in the receiver (different from the other three scenarios, where the sharer appears as transgressor and may signal malintent in the past with no intention to make it right). Possibly, these variables (e.g., the sharer’s role) that go beyond valence may be better suited to predict circumstances under which sharing a secret increases closeness.

### Limitations and future research

Across all three studies, we applied an experimental approach to investigate the effects of sharing secrets on changes in social distance. This approach is associated with many benefits in internal validity such as the possibility to control for variables that may alternatively explain when individuals are more likely to share secrets (e.g., people that are already close may share secrets to further increase closeness, which we controlled for by using standardized materials in which the distance between sharer and receiver was vague). We further made the secret versus information conditions equivalent, meaning that the content shared was identical in both conditions–it was simply labeled as a secret or not. Labelling secrets, in general, ensured that it would be the secretive nature of the information, and not differences in information per se, that drove the effects. This approach, however, also comes with disadvantages. First, labels used in the studies were not identical within a condition (e.g., some contained the word “secret”, others did not) and thereby may have introduced further error variance. Second, the results from Studies 1 and 2 show that negative information not labeled as a secret was perceived as almost as confidential as the negative and positive secrets. This results in a setting that is not ideal to test our hypotheses, as valence and secrecy factors did not seem to be orthogonal. Future research could aim to overcome this limitation by, for example, standardizing labels that identify a piece of information as secret or as nonconfidential and by allowing the content to differ and controlling statistically for differences in intensity or emotionality.

In Studies 1 and 2, the change in social distance was assessed in two ways, directly with a rating on a Likert-scale and with a pictorial measure adapted from the IOS-scale [46]. Both approaches, however, only use one item. Future research could implement more detailed measures of change in social distance that may afford a more nuanced perspective on the effect of secret sharing on relationships.

We further designed Study 3 to test how lay theories about the relational consequences of secret sharing may impact strategic sharing of information. The study scenario (a hypothetical interaction with a student in a seminar), however, might have been too abstract. Participants may simply not want to share personal secrets with a stranger on a first meeting but only after a certain threshold of acquaintance has been passed. Further, our goal manipulation consisted of asking participants to either create closeness (or not) towards the other student. Whether this goal would be reached was neither evaluated nor incentivized–two aspects which might increase the manipulation’s impact on behavior. Future research could work with a more realistic setting (eventually even allow participants to briefly chat or interact with another person) before being asked to share information. The other participant could be allowed to react to the shared information and asked to rate changes in distance to measure whether participants reached their goal. Successfully reaching the goal could moreover be incentivized by offering participants a bonus. Moreover, future research may investigate alternative strategies that may be applied to reach closeness (or distance) goals (e.g., not disclosing any information, disclosing meaningless information).

As described above, future work could also aim to identify and test different predictors that may explain more variance in the effects of secrets on social distance. Across studies, we investigated situations in which one person confides a secret to another and while the content of the secret did in general not involve the recipient directly. Sharing a secret, however, could also entail a confession, meaning a secret that involves the recipient directly [6]. Here, changes in social distance might very much differ and require further investigation. Furthermore, secrets of negative valence may differ regarding agency, vulnerability, malintent, fear of discovery and so on. Previous work has shown that the extent to which secrets are perceived as immoral, relational, and professional/goal-oriented are important dimensions [65]. Future work could investigate which of these variables are best suited to predict changes in social distance, thereby going beyond the valence dimension used in the studies presented here.

### Implications

The here presented research could have implications for both research and the public. For research, results inform on the social nature of secrets and secret sharing. Confiding a secret may result in a decrease in social distance. Results, however, also indicate that this effect might depend on the specific content of the shared information and the sharer’s role; further research might explore these potential moderator variables. For the public, this work may show that confiding in somebody else may be a fruitful strategy to cope when carrying a secret. Sharing a secret with somebody, who is not directly affected by the secret, may allow a new perspective to be gained, see [6], and eventually reduce social distance between sharer and recipient.

### Conclusion

When individuals decide to share a secret, this may not only impact themselves or the receiver, but also the relationship between them. Across studies we show that after a secret is shared, receivers and observers judge the sharer and receiver as closer than before. Furthermore, sharing positive compared to negative content (regardless of whether the content is secret or not) results in more closeness. When sharing information, participants rather prefer to share neutral information than secrets with a stranger. If they share a secret, it is more likely a positive rather than a negative one–whether they were instructed to create closeness or keep distance did not significantly affect this tendency. Our findings speak to the so far neglected social nature of secret sharing and indicate that sharing secrets can reduce the distance between two people and thereby impact their relationship.

## Supporting information

S1 File Vignettes with positive information. (PDF)

S2 File Vignettes with negative information. (PDF)

S3 File (DOCX)
