# Supplementary material for: Secretive and close? How sharing secrets may impact perceptions of distance
Source: PLoS One. 2023 Apr 26;18(4):e0282643. doi: 10.1371/journal.pone.0282643 (PMC10132672; doi:10.1371/journal.pone.0282643)
Supplement: S3 File — (DOCX) [file pone.0282643.s003.docx]

Additionally, anonymized data can be found here: https://osf.io/m68sr/.
